# Bis{2,2′-[(2-amino­eth­yl)aza­nedi­yl]diethanaminium} di-μ-sulfido-bis­(disulfido­germanate)

**DOI:** 10.1107/S160053681200092X

**Published:** 2012-01-14

**Authors:** Chao Xu, Jing-Jing Zhang, Taike Duan, Qun Chen, Qian-Feng Zhang

**Affiliations:** a Institute of Molecular Engineering and Applied Chemistry, Anhui University of Technology, Ma’anshan, Anhui 243002, People’s Republic of China; b Department of Applied Chemistry, School of Petrochemical Engineering, Changzhou University, Jiangsu 213164, People’s Republic of China

## Abstract

In the title compound, (C_6_H_20_N_4_)_2_[Ge_2_S_6_], the dimeric [Ge_2_S_6_]^4−^ anion is formed by two edge-sharing GeS_4_ tetra­hedral units. The average terminal and bridging Ge—S bond lengths are 2.158 (14) and 2.276 (6) Å, respectively. The anions and the diprotonated ammonium cations are organized into a three-dimensional network by N—H⋯S and N—H⋯N hydrogen bonds.

## Related literature

For background to main group metal–chalcogenide compounds, see: Bowes & Ozin (1996 ▶); Zheng *et al.* (2002 ▶, 2005 ▶). For a related structure, see: Jia *et al.* (2005 ▶).
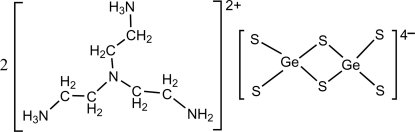


## Experimental

### 

#### Crystal data

(C_6_H_20_N_4_)_2_[Ge_2_S_6_]
*M* *_r_* = 634.06
Monoclinic, 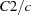

*a* = 25.2845 (17) Å
*b* = 7.3173 (4) Å
*c* = 16.6001 (9) Å
β = 122.637 (4)°
*V* = 2586.3 (3) Å^3^
*Z* = 4
Mo *K*α radiation
μ = 2.83 mm^−1^
*T* = 296 K
0.19 × 0.16 × 0.15 mm

#### Data collection

Bruker APEXII CCD diffractometer
Absorption correction: multi-scan (*SADABS*; Sheldrick, 1996 ▶) *T*_min_ = 0.616, *T*_max_ = 0.677
11988 measured reflections
2952 independent reflections
2243 reflections with *I* > 2σ(*I*)
*R*_int_ = 0.051

#### Refinement

*R*[*F* ^2^ > 2σ(*F* ^2^)] = 0.042
*wR*(*F* ^2^) = 0.111
*S* = 1.03
2952 reflections
159 parameters
H atoms treated by a mixture of independent and constrained refinement
Δρ_max_ = 1.05 e Å^−3^
Δρ_min_ = −1.08 e Å^−3^

### 

Data collection: *APEX2* (Bruker, 2007 ▶); cell refinement: *SAINT* (Bruker, 2007 ▶); data reduction: *SAINT*; program(s) used to solve structure: *SHELXS97* (Sheldrick, 2008 ▶); program(s) used to refine structure: *SHELXL97* (Sheldrick, 2008 ▶); molecular graphics: *SHELXTL* (Sheldrick, 2008 ▶); software used to prepare material for publication: *SHELXTL*.

## Supplementary Material

Crystal structure: contains datablock(s) I, global. DOI: 10.1107/S160053681200092X/hy2497sup1.cif

Structure factors: contains datablock(s) I. DOI: 10.1107/S160053681200092X/hy2497Isup2.hkl

Additional supplementary materials:  crystallographic information; 3D view; checkCIF report

## Figures and Tables

**Table 1:** Hydrogen-bond geometry (Å, °)

*D*—H⋯*A*	*D*—H	H⋯*A*	*D*⋯*A*	*D*—H⋯*A*
N2—H1*N*⋯S3^i^	0.85 (4)	2.61 (5)	3.445 (4)	170 (4)
N2—H2*N*⋯S2	0.73 (8)	2.91 (8)	3.470 (4)	136 (6)
N2—H2*N*⋯S3	0.73 (8)	2.89 (8)	3.514 (4)	145 (7)
N2—H3*N*⋯N3^ii^	0.99 (4)	1.95 (4)	2.897 (5)	159 (4)
N4—H6*N*⋯S3^i^	0.90 (5)	2.44 (5)	3.311 (4)	163 (4)
N4—H7*N*⋯S2^iii^	0.87 (4)	2.50 (4)	3.357 (4)	170 (3)
N4—H8*N*⋯S3^iv^	0.90 (4)	2.47 (4)	3.362 (4)	171 (3)

Symmetry codes: (i) 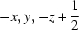; (ii) 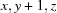; (iii) 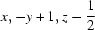; (iv) 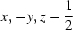.

**Fig. 1.**
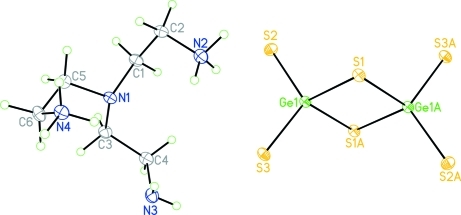
The structure of the title compound, showing displacement ellipsoids at the 50% probability level.

**Fig. 2.**
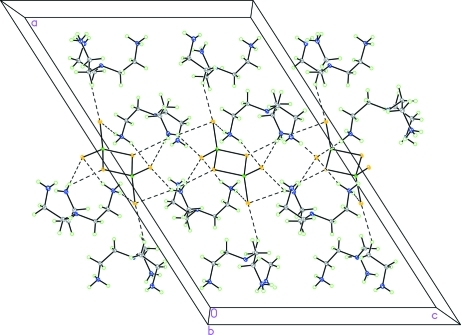
Packing diagram of the title compound. Dashed lines denote hydrogen bonds.

## supplementary crystallographic information

### Comment

There has been an extensive interest in main group metal–chalcogenide compounds because of their unique structures and potential applications in areas such as semiconductors and photocatalysis (Zheng *et al.*, 2005). To synthesize related compounds, many attempts have been made to the reaction of metal-sulfur fluxes at high temperature (Bowes & Ozin, 1996). Compared to the harsh conditions, solvothermal synthesis in a lower temperature is the most efficient choice for the synthesis of metal–chalcogenide complexes (Zheng *et al.*, 2002). In this paper, we report the hydrothermal synthesis and crystal structure of a new thiogermanate, [taeaH_2_]_2_[Ge_2_S_6_] (taea = tris(2-aminoethyl)amine).

The title compound is composed of a dimeric [Ge_2_S_6_]^4-^ anion and two diprotonated [taeaH_2_]^2+^ cations (Fig. 1). The dimeric anion is constructed by two edge-sharing tetrahedral GeS_4_ units, forming a planar four-membered Ge_2_S_2_ ring. The S—Ge—S angles from the tetrahedral unit display a range from 94.68 (3) to 115.75 (4)°. The Ge—S—Ge angle in the four-membered Ge_2_S_2_ ring is 85.32 (3)°. The average bond length of Ge—S_t_ (terminal bond) is shorter than that of Ge—S_b_ (bridging bond) by 0.118 Å. The bond parameters in the title compound are similar to those found in the other thiogermmanates (Jia *et al.*, 2005). Two terminal amine groups from the taea molecule are protonated to balance negative charges of the dimeric anion. The anions and cations are organized into an extended three-dimensional network by N—H···N and N—H···S hydrogen bonds (Fig. 2 and Table 1).

### Experimental

GeO_2_ (104.6 mg, 1.0 mmol) and S (128.0 mg, 4.0 mmol) were mixed with tris(2-aminoethyl)amine (2.0569 g) in a 23 ml Teflon-lined stainless steel autoclave and stirred for 20 min. The vessel was sealed and heated to 190°C for 6 d and then cooled to room temperature. Colorless flake crystals were obtained and air dried. The yield based on GeO_2_ is about 40%. Analysis, calculated for C_12_H_40_Ge_2_N_8_S_6_: C 22.7, H 6.36, N 17.7%; found: C 22.5, H 6.31, N 17.6%.

### Refinement

C-bound H atoms were positioned geometrically and refined as riding atoms. with C—H = 0.97 (CH_2_) Å and with *U*_iso_(H) = 1.2*U*_eq_(C). N-bound H atoms were located from a difference Fourier map and refined isotropically.

### Crystal data

**Table:**

(C_6_H_20_N_4_)_2_[Ge_2_S_6_]	*F*(000) = 1312
*M**_r_* = 634.06	*D*_x_ = 1.628 Mg m^−^^3^
Monoclinic, *C*2/*c*	Mo *K*α radiation, λ = 0.71073 Å
Hall symbol: -C 2yc	Cell parameters from 3750 reflections
*a* = 25.2845 (17) Å	θ = 2.9–26.8°
*b* = 7.3173 (4) Å	µ = 2.83 mm^−^^1^
*c* = 16.6001 (9) Å	*T* = 296 K
β = 122.637 (4)°	Block, colorless
*V* = 2586.3 (3) Å^3^	0.19 × 0.16 × 0.15 mm
*Z* = 4	

### Data collection

**Table:**

Bruker APEXII CCD diffractometer	2952 independent reflections
Radiation source: fine-focus sealed tube	2243 reflections with *I* > 2σ(*I*)
graphite	*R*_int_ = 0.051
φ and ω scans	θ_max_ = 27.5°, θ_min_ = 1.9°
Absorption correction: multi-scan (*SADABS*; Sheldrick, 1996)	*h* = −28→32
*T*_min_ = 0.616, *T*_max_ = 0.677	*k* = −9→9
11988 measured reflections	*l* = −21→20

### Refinement

**Table:**

Refinement on *F*^2^	Primary atom site location: structure-invariant direct methods
Least-squares matrix: full	Secondary atom site location: difference Fourier map
*R*[*F*^2^ > 2σ(*F*^2^)] = 0.042	Hydrogen site location: inferred from neighbouring sites
*wR*(*F*^2^) = 0.111	H atoms treated by a mixture of independent and constrained refinement
*S* = 1.03	*w* = 1/[σ^2^(*F*_o_^2^) + (0.0694*P*)^2^] where *P* = (*F*_o_^2^ + 2*F*_c_^2^)/3
2952 reflections	(Δ/σ)_max_ = 0.001
159 parameters	Δρ_max_ = 1.05 e Å^−^^3^
0 restraints	Δρ_min_ = −1.07 e Å^−^^3^

### Special details

**Table:**

Geometry. All esds (except the esd in the dihedral angle between two l.s. planes) are estimated using the full covariance matrix. The cell esds are taken into account individually in the estimation of esds in distances, angles and torsion angles; correlations between esds in cell parameters are only used when they are defined by crystal symmetry. An approximate (isotropic) treatment of cell esds is used for estimating esds involving l.s. planes.
Refinement. Refinement of F^2^ against ALL reflections. The weighted R-factor wR and goodness of fit S are based on F^2^, conventional R-factors R are based on F, with F set to zero for negative F^2^. The threshold expression of F^2^ > 2sigma(F^2^) is used only for calculating R-factors(gt) etc. and is not relevant to the choice of reflections for refinement. R-factors based on F^2^ are statistically about twice as large as those based on F, and R- factors based on ALL data will be even larger.

### Fractional atomic coordinates and isotropic or equivalent isotropic displacement parameters (Å^2^)

**Table:**

	*x*	*y*	*z*	*U*_iso_*/*U*_eq_	
Ge1	0.046082 (16)	0.40953 (4)	0.47830 (2)	0.02516 (14)	
S1	−0.02392 (4)	0.64364 (11)	0.40812 (6)	0.0284 (2)	
S2	0.14075 (4)	0.50236 (13)	0.53795 (7)	0.0368 (2)	
S3	0.01711 (4)	0.17923 (11)	0.38125 (6)	0.0322 (2)	
N1	0.16919 (14)	0.0976 (3)	0.2936 (2)	0.0291 (7)	
N2	0.10013 (18)	0.4325 (5)	0.3045 (3)	0.0375 (8)	
H1N	0.074 (2)	0.358 (6)	0.263 (3)	0.042 (12)*	
H2N	0.096 (3)	0.399 (9)	0.342 (6)	0.11 (3)*	
H3N	0.0834 (19)	0.559 (6)	0.295 (3)	0.043 (11)*	
N3	0.08225 (18)	−0.1798 (5)	0.3167 (3)	0.0411 (8)	
H4N	0.061 (2)	−0.179 (7)	0.340 (4)	0.062 (17)*	
H5N	0.066 (2)	−0.111 (6)	0.277 (3)	0.040 (14)*	
N4	0.09320 (16)	0.0946 (5)	0.0738 (2)	0.0344 (7)	
H6N	0.070 (2)	0.112 (6)	0.100 (4)	0.054 (14)*	
H7N	0.1009 (18)	0.199 (6)	0.058 (3)	0.038 (11)*	
H8N	0.0691 (18)	0.031 (5)	0.019 (3)	0.034 (10)*	
C1	0.19669 (17)	0.2513 (5)	0.3611 (3)	0.0353 (8)	
H1A	0.2403	0.2653	0.3812	0.042*	
H1B	0.1957	0.2233	0.4174	0.042*	
C2	0.16258 (18)	0.4298 (5)	0.3188 (3)	0.0370 (9)	
H2A	0.1874	0.5293	0.3610	0.044*	
H2B	0.1584	0.4496	0.2578	0.044*	
C3	0.18437 (18)	−0.0753 (5)	0.3472 (3)	0.0375 (9)	
H3A	0.2283	−0.0737	0.3987	0.045*	
H3B	0.1784	−0.1755	0.3048	0.045*	
C4	0.14468 (19)	−0.1096 (5)	0.3891 (3)	0.0396 (9)	
H4A	0.1659	−0.1972	0.4409	0.048*	
H4B	0.1401	0.0035	0.4152	0.048*	
C5	0.19364 (17)	0.0959 (5)	0.2311 (3)	0.0342 (8)	
H5A	0.2350	0.0400	0.2652	0.041*	
H5B	0.1983	0.2209	0.2165	0.041*	
C6	0.15200 (18)	−0.0063 (5)	0.1386 (3)	0.0372 (8)	
H6A	0.1745	−0.0252	0.1070	0.045*	
H6B	0.1419	−0.1252	0.1526	0.045*	

### Atomic displacement parameters (Å^2^)

**Table:**

	*U*^11^	*U*^22^	*U*^33^	*U*^12^	*U*^13^	*U*^23^
Ge1	0.0285 (2)	0.0261 (2)	0.0180 (2)	0.00119 (14)	0.01058 (16)	0.00061 (12)
S1	0.0353 (5)	0.0292 (4)	0.0177 (4)	0.0043 (3)	0.0123 (4)	0.0038 (3)
S2	0.0288 (5)	0.0419 (6)	0.0347 (5)	−0.0023 (4)	0.0138 (4)	0.0004 (4)
S3	0.0412 (5)	0.0293 (5)	0.0231 (4)	0.0015 (4)	0.0153 (4)	−0.0034 (3)
N1	0.0328 (16)	0.0249 (14)	0.0219 (14)	0.0012 (12)	0.0097 (12)	0.0013 (11)
N2	0.045 (2)	0.036 (2)	0.0295 (18)	0.0025 (16)	0.0191 (17)	0.0009 (15)
N3	0.047 (2)	0.043 (2)	0.035 (2)	0.0016 (17)	0.0230 (18)	−0.0023 (16)
N4	0.0356 (18)	0.0358 (18)	0.0248 (16)	0.0000 (15)	0.0116 (15)	0.0001 (14)
C1	0.0354 (19)	0.0322 (19)	0.0274 (18)	−0.0033 (15)	0.0097 (15)	−0.0042 (14)
C2	0.041 (2)	0.0298 (19)	0.037 (2)	−0.0050 (16)	0.0187 (18)	−0.0034 (15)
C3	0.044 (2)	0.0283 (19)	0.039 (2)	0.0064 (16)	0.0216 (18)	0.0079 (15)
C4	0.050 (2)	0.036 (2)	0.029 (2)	−0.0008 (17)	0.0186 (18)	0.0017 (15)
C5	0.0314 (19)	0.038 (2)	0.0273 (18)	0.0032 (15)	0.0119 (16)	0.0041 (14)
C6	0.044 (2)	0.035 (2)	0.0268 (18)	0.0076 (16)	0.0151 (16)	−0.0008 (15)

### Geometric parameters (Å, °)

**Table:**

Ge1—S2	2.1482 (10)	N4—H8N	0.90 (4)
Ge1—S3	2.1677 (9)	C1—C2	1.514 (5)
Ge1—S1^i^	2.2715 (9)	C1—H1A	0.9700
Ge1—S1	2.2804 (9)	C1—H1B	0.9700
N1—C5	1.466 (5)	C2—H2A	0.9700
N1—C1	1.471 (4)	C2—H2B	0.9700
N1—C3	1.473 (4)	C3—C4	1.519 (6)
N2—C2	1.465 (5)	C3—H3A	0.9700
N2—H1N	0.85 (4)	C3—H3B	0.9700
N2—H2N	0.73 (8)	C4—H4A	0.9700
N2—H3N	0.99 (4)	C4—H4B	0.9700
N3—C4	1.467 (5)	C5—C6	1.511 (5)
N3—H4N	0.81 (5)	C5—H5A	0.9700
N3—H5N	0.75 (5)	C5—H5B	0.9700
N4—C6	1.479 (5)	C6—H6A	0.9700
N4—H6N	0.90 (5)	C6—H6B	0.9700
N4—H7N	0.87 (4)		
			
S2—Ge1—S3	115.74 (4)	N2—C2—C1	112.4 (3)
S2—Ge1—S1^i^	112.57 (4)	N2—C2—H2A	109.1
S3—Ge1—S1^i^	110.36 (4)	C1—C2—H2A	109.1
S2—Ge1—S1	111.28 (4)	N2—C2—H2B	109.1
S3—Ge1—S1	110.26 (3)	C1—C2—H2B	109.1
S1^i^—Ge1—S1	94.69 (3)	H2A—C2—H2B	107.9
Ge1^i^—S1—Ge1	85.31 (3)	N1—C3—C4	113.4 (3)
C5—N1—C1	109.8 (3)	N1—C3—H3A	108.9
C5—N1—C3	110.4 (3)	C4—C3—H3A	108.9
C1—N1—C3	109.5 (3)	N1—C3—H3B	108.9
C2—N2—H1N	116 (3)	C4—C3—H3B	108.9
C2—N2—H2N	120 (6)	H3A—C3—H3B	107.7
H1N—N2—H2N	94 (6)	N3—C4—C3	111.6 (3)
C2—N2—H3N	112 (2)	N3—C4—H4A	109.3
H1N—N2—H3N	113 (4)	C3—C4—H4A	109.3
H2N—N2—H3N	101 (5)	N3—C4—H4B	109.3
C4—N3—H4N	108 (4)	C3—C4—H4B	109.3
C4—N3—H5N	109 (3)	H4A—C4—H4B	108.0
H4N—N3—H5N	102 (5)	N1—C5—C6	113.3 (3)
C6—N4—H6N	112 (3)	N1—C5—H5A	108.9
C6—N4—H7N	111 (3)	C6—C5—H5A	108.9
H6N—N4—H7N	109 (4)	N1—C5—H5B	108.9
C6—N4—H8N	110 (2)	C6—C5—H5B	108.9
H6N—N4—H8N	107 (4)	H5A—C5—H5B	107.7
H7N—N4—H8N	107 (4)	N4—C6—C5	111.6 (3)
N1—C1—C2	112.9 (3)	N4—C6—H6A	109.3
N1—C1—H1A	109.0	C5—C6—H6A	109.3
C2—C1—H1A	109.0	N4—C6—H6B	109.3
N1—C1—H1B	109.0	C5—C6—H6B	109.3
C2—C1—H1B	109.0	H6A—C6—H6B	108.0
H1A—C1—H1B	107.8		

Symmetry codes: (i) −*x*, −*y*+1, −*z*+1.

### Hydrogen-bond geometry (Å, °)

**Table:**

*D*—H···*A*	*D*—H	H···*A*	*D*···*A*	*D*—H···*A*
N2—H1N···S3^ii^	0.85 (4)	2.61 (5)	3.445 (4)	170 (4)
N2—H2N···S2	0.73 (8)	2.91 (8)	3.470 (4)	136 (6)
N2—H2N···S3	0.73 (8)	2.89 (8)	3.514 (4)	145 (7)
N2—H3N···N3^iii^	0.99 (4)	1.95 (4)	2.897 (5)	159 (4)
N4—H6N···S3^ii^	0.90 (5)	2.44 (5)	3.311 (4)	163 (4)
N4—H7N···S2^iv^	0.87 (4)	2.50 (4)	3.357 (4)	170 (3)
N4—H8N···S3^v^	0.90 (4)	2.47 (4)	3.362 (4)	171 (3)

Symmetry codes: (ii) −*x*, *y*, −*z*+1/2; (iii) *x*, *y*+1, *z*; (iv) *x*, −*y*+1, *z*−1/2; (v) *x*, −*y*, *z*−1/2.
